# Editorial: How *Salmonella* Infection can Inform on Mechanisms of Immune Function and Homeostasis

**DOI:** 10.3389/fimmu.2015.00451

**Published:** 2015-09-03

**Authors:** Constantino López-Macías, Adam F. Cunningham

**Affiliations:** ^1^Medical Research Unit on Immunochemistry, National Medical Centre “Siglo XXI”, Mexican Institute for Social Security, Specialties Hospital, Mexico City, Mexico; ^2^Institute for Biomedical Research, School of Immunity and Infection, University of Birmingham, Birmingham, UK

**Keywords:** *Salmonella*, host responses, microbiota, innate immune system, adaptive immune system

Our ability to survive requires the competency to control infection. In the last 50 years, there has been an explosion in our understanding of the processes that underlie this. Central to this is our ability to restrict the infection to local sites and so prevent it from becoming systemic. Infections caused by serovars of the species *Salmonella enterica*, spread through fecal–oral transmission, exemplify this and are a major reason why this organism was chosen as the theme of this Research Topic. *Salmonella* infections, particularly typhoidal infections, have had their hand on the tiller of human history, able to steer fate in new directions as a consequence of their deadly properties. A key element of this is the ability to spread through the host and this is often associated with the capacity to cause fatal infections. The prevention of infection and the control of bacterial spread require the complex interplay between the microbiota and innate and adaptive immune mechanisms. The effects of *Salmonella* infections on the complex systems that regulate their control can leave short- and long-term footprints on the homeostatic functions of the host, for instance in the thymus and bone marrow (1, 2), broadens the significance of their study. The depth of interest in this organism is represented in this Research Topic.

The reasons behind the diverse clinical manifestations of this infection are introduced by Gal-Mor and colleagues (3), who discuss the differences between typhoidal and non-typhoidal *Salmonella* strains. This overview includes introducing antigens, including Vi capsule, which can be differentially expressed, as well as the distinct immune responses induced by different serovars. Whilst most groups focus their studies on *Salmonella* infections in mammalian hosts, it should be remembered that many serovars can colonize other organisms too, and indeed this provides a reservoir for most non-typhoidal strains. Wigley highlights the importance of *Salmonella* infection in chickens, both as a source of zoonotic infection, but also as a disease in itself and one of major economic importance (4). Furthermore, we can learn so much from this system, for instance chickens lack lymph nodes, have different MHC and TLR usage, and lack IgG subclasses, so the regulation of the immune response is likely to have multiple unique features. Although the severity of *Salmonella* infections in humans and mice is associated with its systemic spread, there is obviously a close relationship with the gut, well described as “a mucosal pathogen with a systemic agenda” (5). In immunological terms, this is a fascinating relationship to study. In many ways, a primary aim of the mucosal system is to limit inflammation to maintain barrier integrity, whereas systemic immunity often dramatically exploits inflammation to contain infection. This is neatly exploited by non-typhoidal *Salmonella* strains that are commonly associated with gastrointestinal infection and inflammation. In addition, Vi-expressing S. Typhi may also exploit lower levels of mucosal inflammation to help it spread throughout the host (6). Several works in this edition refer to relationship between the pathogen and the gut. Santos examines the three-way relationship between *Salmonella*, the microbiota, and the innate immune system, with a particular emphasis on how the microbiota can buffer against infection (7). Patel and McCormick further develop this concept to encompass details on the ability of *Salmonella* to exploit innate barriers and immune cells via type III secretion systems to establish and cause infections (8). This theme is explored in greater depth from a Salmonella perspective by Hurley and colleagues (9), who provide insights into the repertoire of virulence-associated genes used by *Salmonella* during infection. *Salmonella* pathogenicity islands (SPI) are important foci of genes that contribute to virulence. How expression of genes within these SPI is controlled is incompletely understood, particularly for the regulation of expression by transcriptional factors encoded outside the regions themselves. Guadarrama and colleagues discuss the potential role of the transcriptional global regulator LeuO in this process (10). More details of innate-like immune cells at the gut mucosa, and how they function, are provided by Ussher et al. (11), who describe the known and potential roles of mucosal-associated innate-like T cells in *Salmonella* and other infections. One important mechanism used to control intracellular survival, for instance through sensing for bacteria, is the ubiquitin pathway, control of which is a melee which ensues between the host and pathogen. How the host uses this pathway and how *Salmonella* impacts upon it is the subject of a review by Narayanan and Edelmann (12) and they also describe how understanding these interactions helps improve our comprehension of the ubiquitin pathway in health in addition to disease. The reasons underlying genetic susceptibility to *Salmonella* infections are multiple and complex. In a primary research paper, Khan et al. describe susceptibility loci in a non-standard mouse model derived from wild mice (13). This work characterizes the *immunity to typhimurium 3* (*ity3*) locus in greater detail to understand the nature of resistance to this infection.

When barriers and innate immunity are insufficient to control infection in mice and humans, then adaptive immunity, including T cells and antibody responses, can contribute to pathogen control to varying degrees. A number of papers in this Research Topic examine the role of adaptive immune responses in the generation of immunity to *Salmonella* and different strategies for vaccine development. O’Donnell and McSorley (14) expand our current appreciation of the role of T cells against bacteria. They do this by examining the development of classical T helper 1 responses to bacterial antigens, but also examine innate-like T cell activation and bystander T cell activation. Furthermore, they discuss the roles and importance of these cells and how infection can subvert their activities. CD4 T cells and the Th1 cells are most associated with control of *Salmonella* infections. Nevertheless, CD8 T cells and B cells are likely to be important too. The relationship between these latter two cell types is examined by Lopez-Medina and colleagues (15). They assess whether infection of B cells results in cross-talk between these cells and CD8 T cells and the potential for this to influence the generation of immunity. This has not been widely explored in the literature and may indicate a role of B cells for aiding in the dissemination of infection. A broad assessment of the immune response to typhoid in humans is provided by Sztein and colleagues (16) and covers T and B cell responses to active infection and the major antigens recognized by the host during infection. A key focus of this study and the central point of the article by Jones et al. is the reintroduction of the human challenge model for typhoid (17, 18). The ability to know the exact time an individual is infected overcomes a major complication in the study of this disease, which is identifying the stage after infection a response is being measured. This should help to identify improved ways to diagnose infection and protect against it through vaccination.

Typhoid is unusual in that there are three vaccines that provide similar protection against disease, albeit that the protection is limited and relatively short-lived, reflecting the efforts employed to limit its spread. Bumann, MacLennan, and Sztein et al. (16, 19, 20) examine the mechanisms of protection against infection through vaccination. Sztein et al. describe many studies using live-attenuated vaccines against typhoid, the lessons that have been learned and the potential for conjugate vaccines generated around the Vi antigen. Bumann focuses on identifying the properties of antigens and how to identify those with the potential to make successful subunit vaccines from the many thousands of antigens that constitute this pathogen. Finally, the article from MacLennan also highlights that multiple antigens are likely to be targets of protective antibody and that this can be harnessed for vaccination. Nevertheless, this article also draws attention to the consequences of inappropriate levels of antibody responses that can turn a protective response to one that may actually be detrimental. Collectively, these three contributions highlight the challenges that we face to make effective vaccines to *Salmonella* infections.

This Research Topic has articles that consider the fundamental nature of *Salmonella* infections from the first principles of why one particular serovar causes one infection and a different one a distinct disease, all the way through to the nature of classical immunomodulation of the host through vaccination. Such a spectrum of offerings will help us better understand the nature of this pathogen and how it can control us and how we can control it.

## Conflict of Interest Statement

The authors declare that the research was conducted in the absence of any commercial or financial relationships that could be construed as a potential conflict of interest.
